# Characterization of the complete mitochondrial genome sequence of orangeline wrasse *Halichoeres hartzfeldii* (Bleeker, 1852) with the phylogenetic relationships within the Labridae species

**DOI:** 10.1080/23802359.2019.1669501

**Published:** 2019-09-26

**Authors:** Hua-Yang Guo, Nan Zhang, Ke-Cheng Zhu, Bao-Suo Liu, Liang Guo, Jing-Wen Yang, Dian-Chang Zhang

**Affiliations:** a Division of Aquaculture and Genetic Breeding, South China Sea Fisheries Research Institute, Chinese Academy of Fishery Sciences, Guangzhou, Guangdong, China;; b Key Laboratory of South China Sea Fishery Resources Exploitation & Utilization, Ministry of Agriculture, Guangzhou, Guangdong, China;; c Guangdong Provincial Engineer Technology Research Center of Marine Biological Seed Industry, Guangzhou, Guangdong Province, China

**Keywords:** *Halichoeres hartzfeldii*, mitogenome, phylogenetic analysis

## Abstract

The mitogenome of *Halichoeres hartzfeldii* is 16,481 bp in length. It consists of 37 typical vertebrate mitochondrial genes including 13 protein-coding genes (PCGs), 22 transfer RNA (tRNA), 2 ribosomal RNA (rRNA) genes, and a control region (D-loop). The orientation and arrangement of these genes is similar to that of the other sequenced Labridae mitogenome. The overall base composition of the mitogenome of H. hartzfeldii is biased towards A +T content at 52.9%. Phylogenetic analysis based on 13 concatenated PCGs nucleotide sequences indicated that the genome of *H. hartzfeldii* is closely related to the genus *Halichoeres.*

Orangeline wrasse *Halichoeres hartzfeldii* (Bleeker, 1852) is a marine coral reef fish and belongs to the family Labridae within the suborder Percoidei of order Perciformes, which is widely distributed in the East Indian region to Micronesia and Samoa, southward to Australia and northward to Japan and the South China Sea (To and Shea 2016). The family Labridae are a diverse group of coral reef fish, which are traditionally classified in the suborder Labroidei with the families Labridae, Cichlidae, Embiotocidae, Pomacentridae, Odacidae, Scaridae, and Scaridae (Westneat and Alfaro 2005). The phylogenetic relationships among these groups have been reported (Westneat and Alfaro 2005; Phillips et al. 2016). However, the accuracy of the labrid phylogeny is still a major question. To better understand the structure of the wrasse fish mitogenome and its classification status, we sequenced the mitogenome of *H. hartzfeldii* and used the results in a phylogenetic analysis of Labridae.

The specimen used to sequence the mitogenome of the orangeline wrasse originated from a wild individual, collected by ‘Nanfeng’ expedition ship of South China Sea Fisheries Research Institute, CAFS in the South China Sea of China (20°29.7′ N, 117°56.3′ E). Total genomic DNA was isolated from the muscle tissue. DNA shotgun library of *H. hartzfeldii* mtDNA was constructed and sequenced following the method of Zhang et al. (2015). For genome annotation and genome sequence analysis, Zhu et al. (2017) were referred to. All the specimens (Accession number: Ssfri-F0085) and the genomic DNA were deposited in Tropical and Subtropical Marine Life Museum of South China Sea Fisheries Research Institute, CAFS, Guangzhou city, Guangdong Province, China.

The mitochondrial genome of *H. Hartzfeldii* (GenBank accession no. KY115688.1) is 16,481 bp length and contains 13 protein-coding genes, 22 tRNA genes, 2 rRNA genes, and a control region (D-Loop). Among those genes, 2 rRNAs, 12 PCGs, and 15 tRNAs are encoded on the plus strand (H strand) and the remaining are encoded on the minus strand (L strand). The organization and location of the mitochondrial genome of *H. Hartzfeldii* are in accordance with the pattern observed in the mitogenomes of other typical Labridae (Qi et al. 2013). The overall base composition of the mitogenome of *H. hartzfeldii* is biased towards A + T content at 52.9%. Except for *COXI* and *ND4* (GTG), 11 PCGs start with typical ATN codons (ATG). Five genes (*ND1*, *ATP8*, *ND6*, *ND4L*, and *COXI*) use TAA as the stop codon and seven genes (*ND2*, *ND3*, *ND4*, *ATP6*, *COXII*, *COXIII*, and *Cyt-b*) end with an incomplete stop codon (a terminal T or TA), while AGA stop codon is present in *ND5* gene, which is different from other Labridae mitochondrial genomes (Han et al. 2016).

The phylogenetic relationship of *H. hartzfeldii* and other 15 Labridae species was inferred using the maximum-parsimony (MP) method with MEGA 6.0 (Tamura et al. 2013). The phylogenetic tree shows that *H. hartzfeldii* and *Parajulis poecilepterus* shared a more recent common ancestor than any other species of the tree (Figure 1). The results of phylogenetic trees agreed with previously published phylogenies based on other genetic information (Phillips et al. 2016). This mitochondrial genome provides important resources for addressing taxonomic issues and studying molecular evolution.

## Figures and Tables

**Figure 1.**
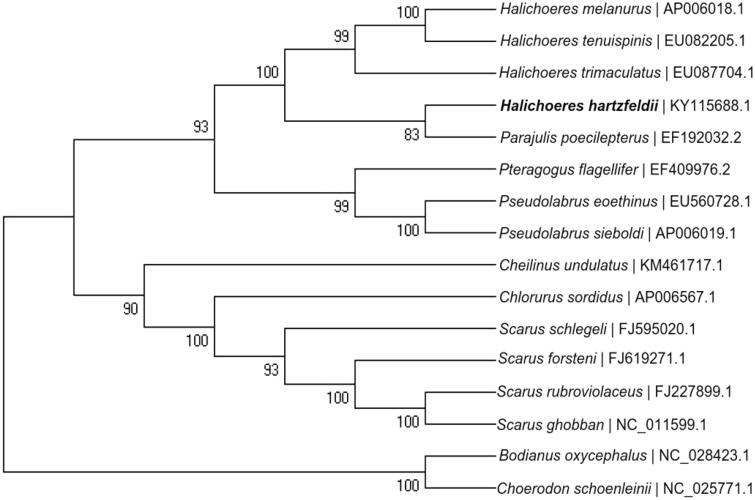
Phylogenetic tree of 16 species within the family Labridae based on the concatenated nucleic acid sequences of 13 PCGs. Numbers beside internal branches indicate MP bootstrap probabilities (BPs) from 1000 replicates (shown as percentages).
